# Fetuin-A: a potential molecular link between obesity, diabetes (type 2 and type 1) and metabolic steatotic liver disease (MASLD)

**DOI:** 10.1007/s40618-026-02880-w

**Published:** 2026-05-04

**Authors:** Ilaria Milani, Gabriele Ciasca, Marianna Chinucci, Valeria Carnazzo, Frida Leonetti, Umberto Basile, Danila Capoccia

**Affiliations:** 1 Department of Medico-Surgical Sciences and Biotechnologies, Faculty of Pharmacy and Medicine, University of Rome La Sapienza, Latina, Lazio, 04100 Italy; 2 Department of Neuroscience, Physics Section, Catholic University of Sacred Heart, Rome, Lazio, 00168 Italy; 3 Department of Clinical Pathology, Ospedale Santa Maria Goretti, Latina, Lazio, 04100 Italy

**Keywords:** Fetuin-A, Non-invasive assessment, Obesity, MASLD, Fibrosis, Type 1 diabetes, Type 2 diabetes

## Abstract

**Purpose:**

Fetuin-A represents a novel molecular target involved in the complex pathogenesis of metabolic disorders. This study aimed to evaluate its association with obesity, type 2 (T2DM) and type 1 (T1DM) diabetes and its correlation with non-invasive liver assessment.

**Methods:**

105 patients (38 with obesity without T2DM, 30 with T2DM, and 37 with T1DM) and 13 controls were included. All participants underwent transient elastography (TE) with controlled attenuation parameter (CAP), and liver stiffness measurement (LSM), clinical and biochemical data (including fetuin-A).

**Results:**

Fetuin-A was significantly higher in all clinical groups than controls, with the greatest increase observed in obesity without T2DM. Fetuin-A correlated positively with measures of adiposity (BMI, waist circumference, waist to height ratio), triglycerides, and non-invasive indicators of liver steatosis (Fatty Liver Index and CAP), while showing no association with fibrosis (defined by LSM ≥ 7.9 kPa). In age- and sex-adjusted models, fetuin-A remained independently associated with obesity and T1DM, whereas T2DM showed a negative but not significant association. Fetuin-A was significantly higher in steatosis, with good discriminatory ability (AUC 0.84, 95% CI 0.77–0.91) and a sensitivity of 0.75 and specificity of 0.74 at the optimal Youden threshold. Negative association with male sex and positive association with age was also observed.

**Conclusions:**

Results confirm fetuin-A as a molecular signature of metabolic disorders, mediating the cross-talk between liver and adipose tissue. Further studies are needed to validate it as a useful biomarker for the early diagnosis and monitoring of liver and metabolic disease, including obesity, T2DM, T1DM, and MASLD, and to reach a consensus on reference values definition.

**Supplementary Information:**

The online version contains supplementary material available at 10.1007/s40618-026-02880-w.

## Introduction

Adipose tissue dysfunction and metabolically associated steatotic liver disease (MASLD) play a central role in the development of insulin resistance, partly through the altered secretion of adipokines and hepatokines [1]. Among these, fetuin-A, also known as α2-Heremans-Schmid glycoprotein (AHSG), is a protein that is predominantly synthesized by the adult liver and partly by adipose tissue which is implicated in several metabolic and inflammatory pathways [2], including insulin signaling impairment, activation of toll-like receptor 4 (TLR4), recruitment and polarization of macrophages, adipocyte dysfunction, hepatic triacylglycerol accumulation, and promotion of liver inflammation and fibrosis [3]. The involvement of metabolic conditions, such as visceral obesity and type 2 diabetes mellitus (T2DM), in MASLD and its progression [4] suggests that fetuin-A may play a regulatory role in the bidirectional relationship between metabolic dysfunction and MASLD, beyond genetic susceptibility [5]. Transient elastography (TE) using FibroScan^®^ is a non-invasive method widely used to assess and stage MASLD by measuring liver stiffness (LSM) and controlled attenuation parameter (CAP) [4]. However, the role of fetuin-A in the adipose-liver crosstalk, as reflected by these parameters, has not been fully elucidated.

Although insulin resistance is a hallmark of T2DM, its contribution to type 1 diabetes mellitus (T1DM) is less well known. In T1DM, Fetuin-A production has been associated with liver insulin resistance [6], poor glycemic control, and its complications [7]. In addition, the increase in MASLD in individuals with T1DM, particularly with overweight [8], highlight the need to further investigate the role of fetuin-A in this population, especially in the context of overweight/obesity.

Recent evidence supports the need for large studies combining hepatokine profiling, imaging, and metabolic assessment to advance precision hepatology [9]. Despite its potential as a biomarker for several metabolic diseases, the clinical application of fetuin-A remains limited by methodological challenges, including the lack of standardized reference values, units and variability among commercial ELISA assays [10–13]. This has led to inconsistency in circulating fetuin-A levels in the literature [10, 11], limiting comparisons between serum fetuin-A measurements to absolute rather than relative, terms and clinical usefulness [12].

This study aims to investigate the relationship between fetuin-A and obesity, T2DM and T1DM and to analyze its correlation with clinical parameters related to metabolic syndrome (MetS) and liver. It will also evaluate the utility of fetuin-A in the diagnosis of MASLD and its severity in these patients as assessed by FibroScan^®^.

### Materials e methods

### Study design and participants

This observational case-control study enrolled 105 patients (38 with obesity without T2DM, 30 with T2DM, and 37 with T1DM) followed at the Diabetes and Obesity Clinic of the Santa Maria Goretti Hospital in Latina, from January 2024 to June 2025 (observation group) and a control group of 13 healthy individuals undergoing routine medical examinations. FibroScan^®^ was used to assess MASLD and divided into steatosis and fibrosis groups. Exclusion criteria included the presence of concomitant chronic liver disease (e.g.,viral, drug-induced liver injury, autoimmune hepatitis, hemochromatosis, primary sclerosing cholangitis, primary biliary cholangitis), hepatocellular carcinoma or other malignancies, excessive alcohol consumption, and pregnancy.

### Anthropometric assessment

Anthropometric measurements, including height (Ht), body weight (BW), and waist circumference (WC), were obtained during the clinical visit. Body mass index (BMI) was calculated as BW (kg) divided by Ht squared (m^2^). WC was measured at the level of the umbilicus with the patient standing upright, using a non-elastic measuring tape. The waist to height ratio (WHtR) was calculated as WC (cm) divided by Ht (cm).

### Laboratory analysis

After an overnight fast of at least 10 h, venous blood samples were obtained from all participants for biochemical analysis. Measurements included triglycerides (TG), total cholesterol (TC), high-density lipoprotein cholesterol (HDL-C), fasting plasma glucose (FPG), alanine aminotransferase (ALT), aspartate aminotransferase (AST), gamma glutamyl transferase (GGT), serum creatinine, C-reactive protein (CRP), fasting insulin, and uric acid. Albuminuria was assessed as the ratio of urinary albumin to creatinine in morning urine samples using a nephelometric method. All biochemical parameters were quantified in the central laboratory of the participating hospital according to standard procedures using proprietary reagents on a fully automated analyzer (Abbott Architect c16000; Abbott Diagnostics, USA). Hemoglobin A1c (HbA1c) was measured by high-performance liquid chromatography (Lifotronic Technology Co., Ltd, Shenzhen, China). Low-density lipoprotein cholesterol (LDL-C) was calculated using the Friedewald formula. Estimated glomerular filtration rate (eGFR) was recorded from the clinical records as calculated using the Modification of Diet in Renal Disease (MDRD). For greater accuracy, eGFR was subsequently recalculated using the Chronic Kidney Disease Epidemiology Collaboration equation (CKD-EPI) [14]. Serum fetuin-A concentrations were determined using a commercially available ELISA kit (Human AHSG ELISA Kit, Antibodies.com, Stockholm, Sweden) and expressed in optical density (OD) units. Consistent with previous work [15, 16], in the absence of standardized reference ranges for Fetuin-A, data were then standardized using z-scores calculated from the distribution of the study cohort for armonization.

### Non-invasive liver disease assessment (NILDAs)

The FLI was calculated using the established formula [17]: [e^(0,953 × ln(TG) + 0,139 × BMI + 0,718 × ln(GGT) + 0,053 × WC − 15,745)] / [1 + e^(0,953 × ln(TG) + 0,139 × BMI + 0,718 × ln(GGT) + 0,053 × WC − 15,745)] × 100.

FIB-4 was determined by using the following formula: age [years] x AST [U/L]/(platelet count [10^9^/L] x √(ALT [U/L])). Age-specific cut-offs were applied as recommended by the MASLD guidelines for descriptive stratification of liver fibrosis risk: a cut-off of 1.3 was used for patients aged ≤ 65 years, and a cut-off of 2.0 was used for patients aged > 65 years [4].

TE using the FibroScan^®^ Compact 530 was performed in the supine position with the right arm abducted. LSM and CAP expressed in kilopascals (kPa) and decibels per meter (dB/m), respectively, were measured in the right hepatic lobe by trained operators. Examinations with ≥ 10 valid measurements and an interquartile range-to-median ratio < 30% were considered reliable. Liver steatosis and fibrosis were defined as CAP ≥ 248 dB/m and LSM ≥ 7.9 kPa, respectively [4].

### Lifestyle and other variables

Age, sex, T2DM and hypertension, and use of antihyperglycemic medications were collected via oral interview. T2DM was defined following the American Diabetes Association (ADA) diagnostic criteria [18]. Hypertension was defined as systolic ≥ 140 mmHg and/or diastolic ≥ 90 mmHg blood pressure or antihypertensive medication. Alcohol consumption was assessed using the Alcohol Use Disorders Identification Test (AUDIT) questionnaire, with a score < 8 indicating low-risk drinking [4].

### Ethics statement and declaration on generative AI in the writing process

The study was conducted in accordance with Helsinki Declaration, and approved by the “Comitato Etico Lazio 1”, the Ethics Committee of the Lazio Region (reference no. 7421, protocol code 0038/2024 and date of approval 10/01/2024). Written informed consent was obtained from all participants.

During the preparation of this manuscript, the authors used ChatGPT (version GPT-4, OpenAI, San Francisco, CA, USA) for the text summary and revision. The authors have reviewed and edited the output and take full responsibility for the content of this publication.

### Statistical analysis

Statistical analyses were performed using R software (version 4.5.2), SPSS software (version 21.0), and OriginPro 2022. For continuous variables, normality was assessed using the Shapiro-Wilk test, and several biomarkers deviated from a Gaussian distribution. Continuous variables were reported as median and interquartile range (IQR), while categorical variables were reported as counts and percentages. Tabular data were generated using the gtsummary package in R. Group comparisons for continuous variables were performed using the Wilcoxon-Mann-Whitney test for two groups or the Kruskal-Wallis test for more than two groups, followed by Dunn’s post hoc test when appropriate. Categorical variables were compared using chi-squared or Fisher’s exact test. Linear and logistic regression analyses were performed in R using the lm and glm functions, respectively, and model assumptions were assessed by testing for residuals and homoscedasticity. The diagnostic performance of fetuin-A for steatosis was assessed by receiver operating characteristic (ROC) curves and area under the curve (AUC) with 95% confidence intervals calculated by DeLong’s method using the pROC package. Correlation analyses were performed using Spearman’s rank correlation. Age- and sex-adjusted partial correlations were calculated using the ppcor package, with confidence intervals estimated by Fisher’s z-transformation, and p-values corrected for multiple testing using the Benjamini-Hochberg false discovery rate procedure.

## Results

### Altered levels of fetuin-A in obesity, T2DM, and T1DM

**Fig. 1:**
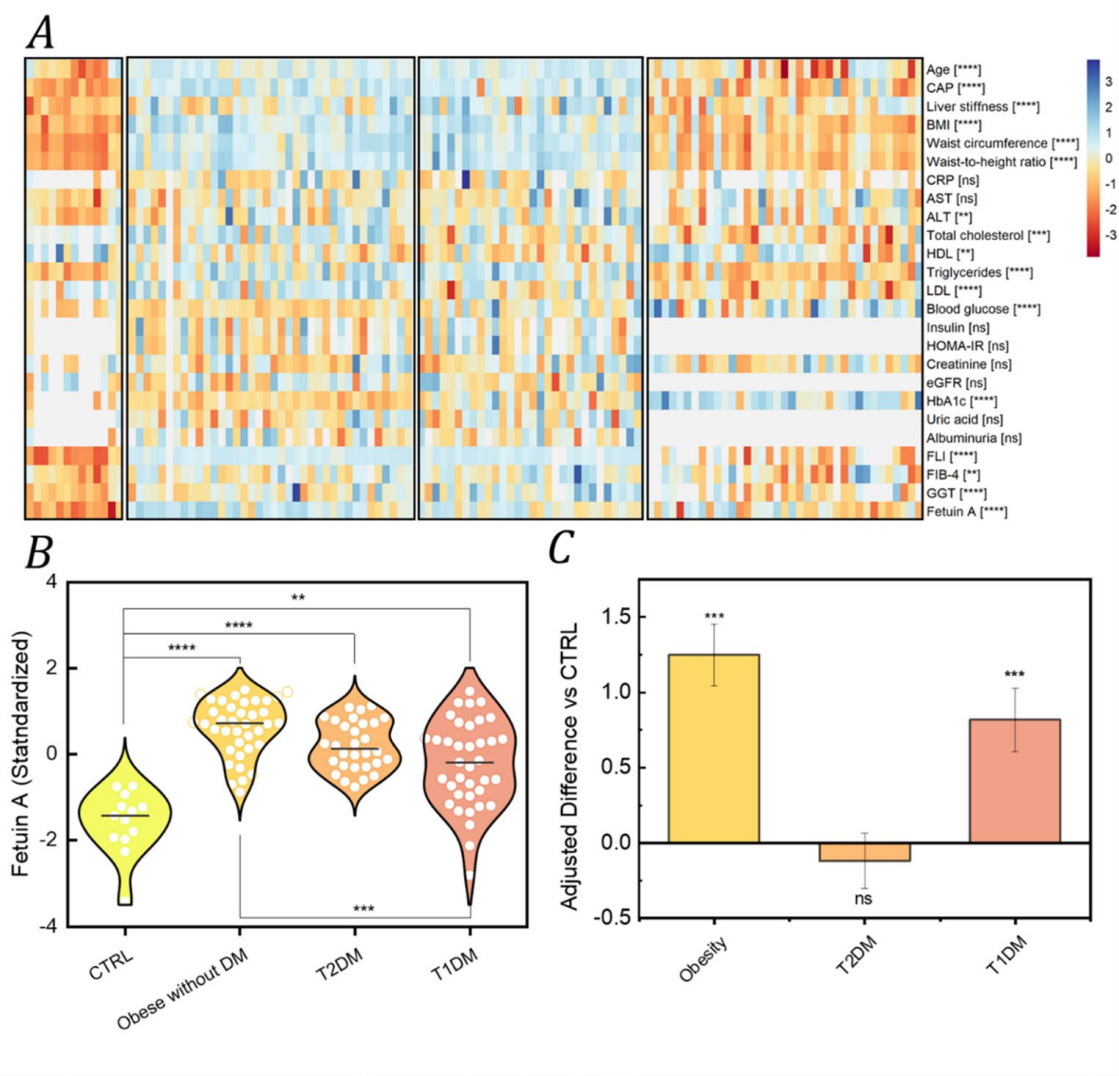
(**A**) Heatmap of continuous variables (anthropometric, lipid, glycemic, renal, and liver-related markers) for individual participants, grouped as controls, obesity without T2DM, T2DM, and T1DM. Values are standardized (z-scores); the color scale indicates relative decrease (blue) or increase (red). Bracketed symbols next to variable names indicate the global Kruskal-Wallis comparison between groups (ns, *, **, ***, ****). (**B**) Groupwise distributions of fetuin-A z-scores shown as violin plots with single observations; horizontal brackets indicate pairwise Dunn-Bonferroni comparisons. (**C**) Multivariable linear model for fetuin-A: adjusted differences versus controls for obesity, T2DM, and T1DM after adjustment for age and sex; bars show estimates with 95% confidence intervals. Abbreviations: WHtR, waist-to-height ratio; CAP, controlled attenuation parameter; LSM, liver stiffness measurement; FLI, Fatty Liver Index; FIB-4, Fibrosis-4; GGT, γ-glutamyl transferase

General characteristics of the whole population are shown in Table S1, Fig. 1A and Figure S1. No statistically significant differences in sex distribution were observed, whereas age differed between groups, with the control and T1DM groups being younger than the others. Obesity without T2DM and T2DM groups had similar anthropometric, liver, and lipid profiles, with the exception of significantly higher HbA1c (6.4 [5.9, 7.2] vs. 5.9 [5.5, 6.0], *p* < 0.001), GGT (32 [22, 46] vs. 24 [19, 28], *p* < 0.001), LSM (6.55 [5.20, 7. 40] vs. 5.55 [4.30, 6.40], *p* = 0.03), and a trend toward a higher percentage of LSM ≥ 7.9 kPa (23.3% vs. 8.6%, *p* = 0.06) in the T2DM groups. Furthermore, nearly all T2DM participants were living with obesity (90%) (Figure S2; Table S1) and a substantial proportion of individuals with obesity without T2DM (approximately 75%) had HbA1c values in the prediabetic range (HbA1c ≥ 5.7%). Controls and T1DM are similar for many characteristics, with the exception of significantly higher FBG and HbA1c in the T1DM groups (119 [97, 160] vs. 86 [80, 98]; 7.65 (6.80, 8.15) vs. 5.50 [5.25, 6.20], *p* < 0.001).

The distributions of fetuin-A (Fig. 1B) showed significantly higher levels in all cases compared to controls, with the greatest levels in the obesity without T2DM group, and a not significant trend toward lower levels in T2DM. Fetuin-A was significantly higher in the T1DM groups than in the control group. Interestingly, T1DM has a more heterogeneous distribution with fewer cases of obesity but the highest percentage of overweight and more than half of the patients exceeding the established risk cut-offs for WC and WHtR (Figure S2; Table S1).

To attempt to separate these effects on circulating fetuin-A, a multivariable linear model including sex, age, obesity, T1DM, and T2DM was performed (Table 1), showing a significant negative association with male sex, and a positive association with age. After adjustment for age and sex, obesity and T1DM remained positively associated with fetuin-A (*p* < 0.001), whereas T2DM showed a negative but not significant association. These adjusted effects with 95% CIs are displayed in Fig. 1C. Results were consistent in sensitivity analyses using BMI as a continuous covariate instead of the obesity indicator. Interaction terms (e.g., obesity × T2DM, age × sex) were not significant, and nested model comparisons (F-tests/likelihood-ratio tests) did not support the inclusion of interactions or additional parameters given the sample size, favoring the more parsimonious models.

**Table 1:** Multivariable linear regression for circulating fetuin-A (z-scores). Model: fetuin-Ai = β0​+β1​⋅Male + β2​⋅Age (years)+β3​⋅T1DM​+β4​⋅T2DM​+β5​⋅Obesity​+ε​. Male, T1DM, T2DM and Obesity are dummy variables (1 = present, 0 = absent; Obesity defined as BMI ≥ 30 kg/m²). The reference profile is female, non-obese, without diabetes; coefficients therefore represent adjusted mean differences in fetuin-A relative to this reference (age effect per 1-year increase). SE = standard error. Significance: *p* < 0.05 (), *p* < 0.01 (), *p* < 0.001 (). Model fit to context: adjusted R^2^ ≈ 0.4; residual SE ≈ 0.77; F(5,112) = 13.89 F(5,112) = 16.8, *p* = 2.3 × 10⁻^12^. T1DM, type 1 diabetes mellitus; T2DM, type 2 diabetes mellitus

Characteristic	Beta^1^	SE
**(Intercept)**	-2.0***	0.387
**Sex (male)**	-0.54**	0.163
**Age (years)**	0.02**	0.007
**T1DM**	0.81***	0.21
**T2DM**	-0.12*	0.184
**Obesity**	1.2***	0.205

Abbreviations: CI = Confidence Interval, SE = Standard Error

^*1*^ *p<0.05; **p<0.01; ***p<0.001

### Correlation of fetuin-A with clinical and metabolic parameters

**Fig. 2:**
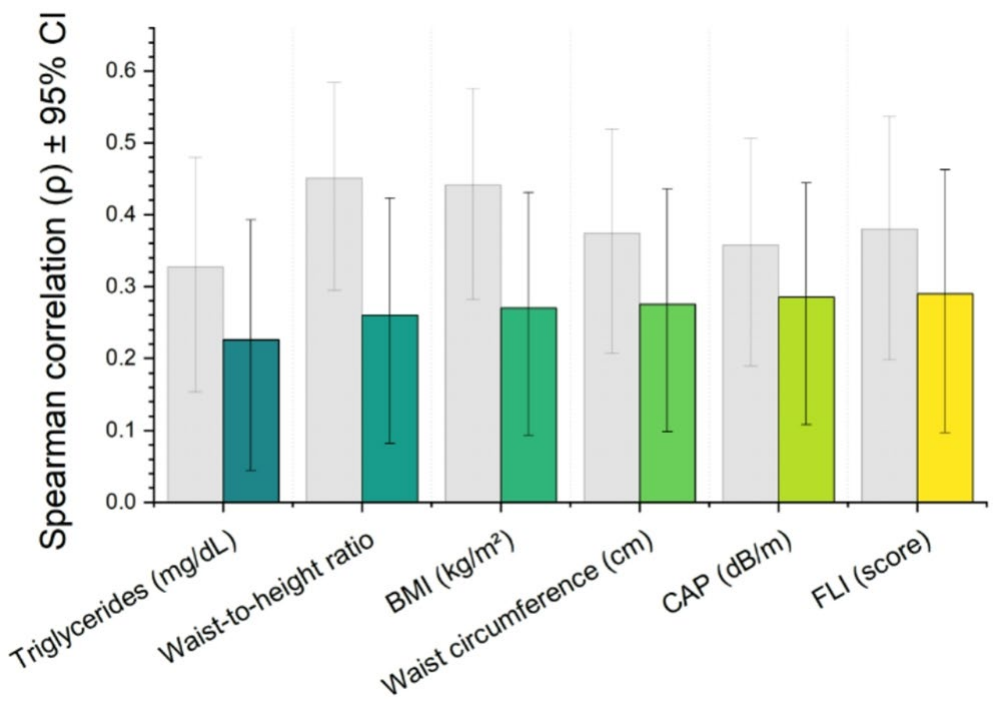
Correlation analysis of circulating fetuin-A with clinical and metabolic variables. Bars represent Spearman correlation coefficients (ρ) with 95% confidence intervals. Unadjusted correlations are shown in grey, while age- and sex-adjusted correlations are shown using the viridis color scale. CAP = controlled attenuation parameter; FLI = fatty liver index

Correlation of fetuin-A with clinical and metabolic variables by calculating Spearman coefficients (ρ) showed a positive correlation with adiposity measures (BMI, WC, WHtR), triglycerides, and with non-invasive steatosis tools (CAP and FLI). The correlation was robust in both unadjusted (ρ = 0.36, 95% CI: 0.19–0.51, *p* < 0.0001) and adjusted analyses (ρ = 0.29, 95% CI: 0.11–0.44, *p* = 0.002) (Table 2; Fig. 2). Interestingly, no correlation was found with liver fibrosis as assessed by FibroScan^®^.

To assess the robustness of all the displayed partial correlations shown, we fitted linear regressions with fetuin-A as the outcome and age, sex, and the corresponding predictor as explanatory variables (data not shown). All coefficients retained in Fig. 2 remained significant, and the models were globally significant. Moreover, all correlations remained robust after controlling for the false discovery rate (FDR) (Table 2).

**Table 2:** Spearman correlations of circulating fetuin-A with clinical and metabolic parameters, reported both unadjusted and adjusted for age and sex (partial ρ). For each variable, correlation coefficients (ρ) are shown with 95% confidence intervals and corresponding raw and FDR-adjusted p-values. Only variables that remained significant in both unadjusted and adjusted analyses are reported. FLI = fatty liver index; CAP = controlled attenuation parameter; WHtR = waist-to-height ratio

Variable	*n*	ρ [95% CI]	*p* (raw)	*p* (FDR)	Partial ρ [95% CI]	*p* (adj)	*p* (FDR adj)
FLI (score)	99	0.38 [0.198, 0.537]	1.04 × 10⁻⁴	1.25 × 10⁻⁴	0.29 [0.097, 0.463]	**0.00390**	**0.00571**
CAP (dB/m)	118	0.358 [0.190, 0.506]	6.76 × 10⁻⁵	1.01 × 10⁻⁴	0.285 [0.108, 0.444]	**0.00194**	**0.00571**
Waist circumference (cm)	118	0.374 [0.207, 0.519]	3.07 × 10⁻⁵	6.15 × 10⁻⁵	0.275 [0.098, 0.436]	**0.00279**	**0.00571**
BMI (kg/m²)	118	0.441 [0.282, 0.576]	5.89 × 10⁻⁷	1.77 × 10⁻⁶	0.270 [0.093, 0.431]	**0.00334**	**0.00571**
Waist-to-height ratio (WHtR)	118	0.451 [0.295, 0.584]	2.89 × 10⁻⁷	1.74 × 10⁻⁶	0.260 [0.082, 0.423]	**0.00475**	**0.00571**
Triglycerides (mg/dL)	117	0.327 [0.154, 0.480]	3.25 × 10⁻⁴	3.25 × 10⁻⁴	0.226 [0.044, 0.393]	**0.0153**	**0.0153**

### Fetuin-A as a potential circulating marker of liver steatosis

**Fig. 3:**
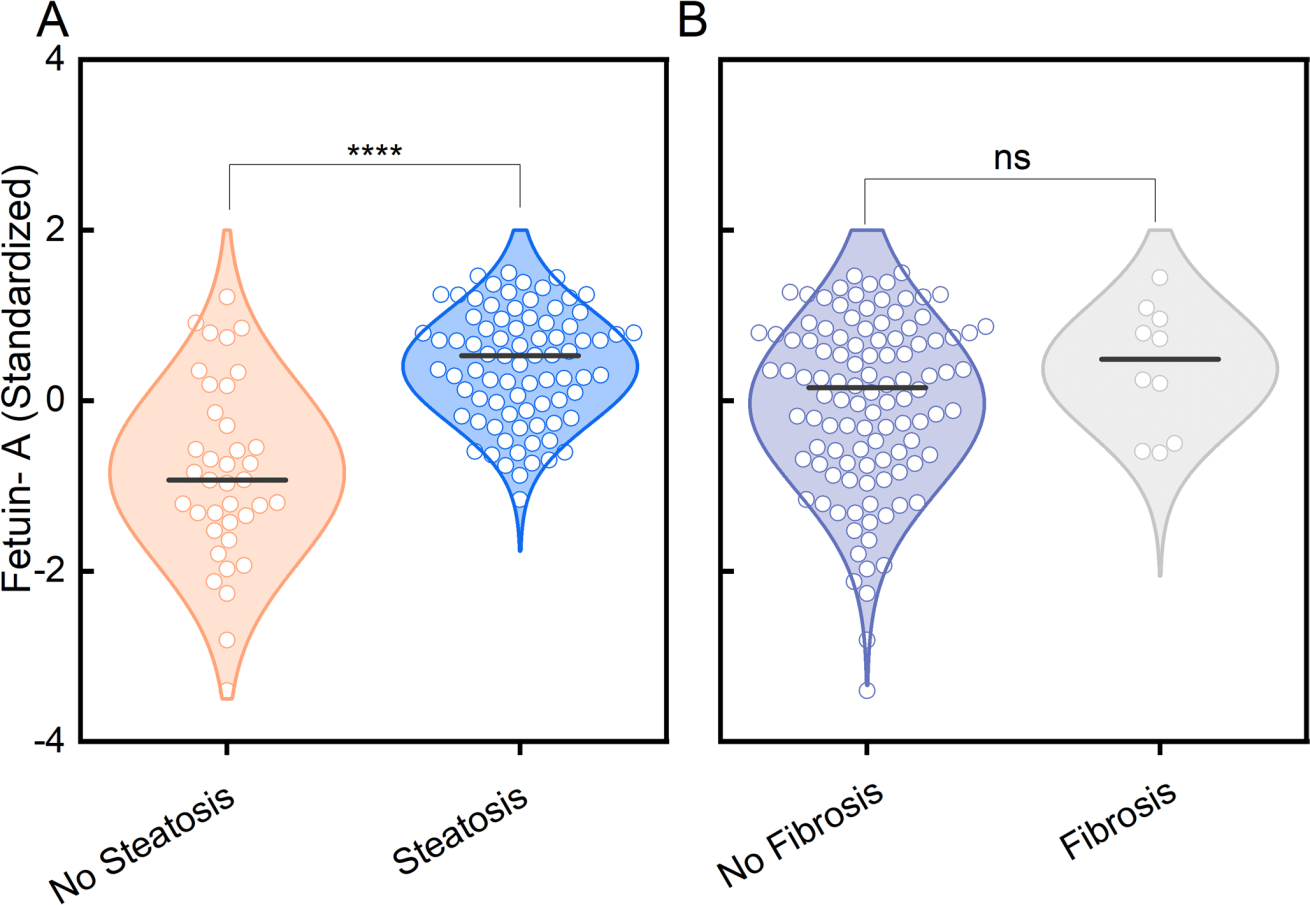
Distribution of circulating fetuin-A levels according to FibroScan ^®^-derived hepatic features. (**A**) Fetuin-A z-scores were significantly higher in individuals with steatosis compared with those without steatosis (Wilcoxon–Mann–Whitney *p* < 0.001). (**B**) No significant differences in fetuin-A z-scores were observed between individuals with and without fibrosis, as defined by liver stiffness measurements (ns)

Consistent with the association of fetuin-A with non-invasive markers of liver steatosis CAP and FLI (Fig. 2; Table 2), the distribution of fetuin-A z-scores showed significantly higher values in those with steatosis, with a clear separation between the two groups and an overall upward shift in the distribution among those with steatosis. Participants without steatosis were largely clustered at values below the cohort mean, whereas those with steatosis had a broader distribution centered around zero or positive z-scores, supporting the ability of fetuin-A to discriminate on the basis of steatosis (Fig. 3A). In contrast, consistent with the lack of association between fetuin-A and liver fibrosis, z-scores did not differ based on significant fibrosis (Fig. 3B).

**Fig. 4:**
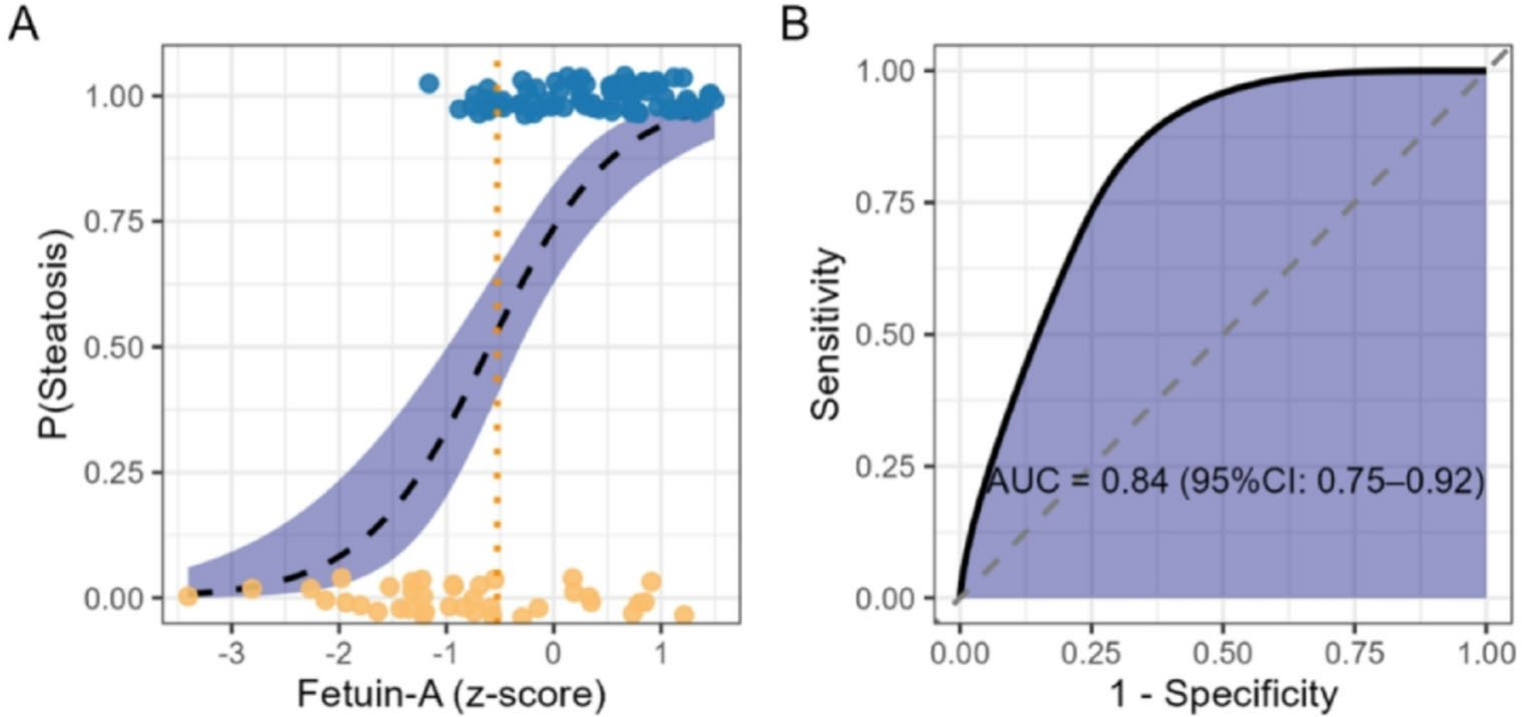
(**A**) Predicted probability of steatosis from a univariable logistic regression model using fetuin-A z-scores as predictor (dashed line), with 95% confidence bands (shaded). Points represent individual participants (orange = no steatosis; blue = steatosis). The vertical reference line marks the optimal Youden point. (**B**) ROC curve for discriminating steatosis using fetuin-A z-scores (AUC 0.84; 95% CI 0.75–0.92)

To further assess the ability of fetuin-A to classify steatosis, we fitted a univariable logistic regression model using fetuin-A z-scores as a predictor. Fetuin-A showed a strong positive association with steatosis (β = 1.72 ± 0.33; *p* = 2.0 × 10⁻⁷), corresponding to an approximately 5.6-fold increase in the odds of steatosis for each 1-unit increase in the fetuin-A z-score (Table 3). The fitted probability curve with 95% confidence bands (Fig. 4A) showed a clear monotonic increase in predicted risk and a clean visual separation between individuals with and without steatosis, with narrow bands consistent with the strength of the association.

**Table 3:** Univariable logistic regression model for steatosis using fetuin-A z-scores as predictor

Term	β (SE)	OR per 1-unit increase in z-score	95% CI for OR	*p*-value
*Intercept*	*1.031 (0.261)*	*—*	*—*	***7.7 × 10⁻⁵***
*Fetuin-A*	*1.721 (0.331)*	*5.60*	*2.92 – 10.70*	***2.1 × 10⁻⁵***

Given this fit, we evaluated the discriminative performance using ROC analysis (Fig. 4B). The ROC curve showed an AUC of 0.84 (95% CI 0.75–0.92). Because z-scores are inherently cohort-dependent and lack clinically interpretable units, no operational cut-off was derived; the vertical reference line in Fig. 4A marks the optimal Youden point, at which fetuin-A achieved a sensitivity of 0.75 and a specificity of 0.74.

## Discussion

Growing evidence supports fetuin-A as a key molecular target in metabolic disorders [19] and an emerging biomarker for MASLD [3]. However, the lack of standardized reference ranges results in high inter-study variability [11], hindering its implementation as a clinically useful biomarker for metabolic diseases [10–12].

In our study, fetuin-A: (a) was significantly increased in all cases compared to controls, with the highest levels in obesity without T2DM,, followed by the T2DM and T1DM groups; (b) was positively correlated with measures of adiposity, TG, FLI and CAP; (c) remained positively associated with obesity and T1DM after sex and age adjustment, while showing a negative not-significant association with T2DM; (d) was significantly higher in steatosis assessed by FibroScan ^®^, while no significant differences were observed with/without fibrosis; (e) was confirmed as a strong predictor of steatosis by logistic regression analysis, showing an approximately 5.6-fold increase in the odds of steatosis, and a higher predictive ability (AUC = 0.84); (f) showed a negative association with male sex and a positive association with age.

Significantly higher Fetuin-A levels found in obesity without T2DM, followed by T2DM and T1DM, and its significant correlations with MetS characteristics and non-invasive steatosis assessment further support its potential as an early diagnostic biomarker for obesity and related metabolic complications, including MASLD [2, 3].

Evidence suggests that liver is the main source of fetuin-A production in adults (> 95%), while its expression in other tissues remains unclear. A small contribution from adipocytes has been reported [2], with increased secretion observed in visceral and subcutaneous fat, especially in the presence of obesity [20]. However, some studies have observed increased fetuin-A protein in adipose tissue of people with obesity and diabetes, despite the absence of mRNA transcripts [21, 22]. This has led to the hypothesis that adipose tissue may act as a reservoir, essentially a “sponge” for liver-derived fetuin-A, sequestering most, if not all, of the protein released into the circulation and thereby providing temporary protection [21, 22]. Thus, we can hypothesize that, in our study, the strong association between fetuin-A and all measures of adiposity, and the higher levels in all cases than in controls, may in part reflect the accumulation of liver-derived fetuin-A in adipose tissue rather than its production by fat. Indeed, according to FLI ≥ 60 and/or CAP ≥ 248 dB/m, liver steatosis was confirmed in all/almost all patients with obesity with/without T2DM, as well as in more than half/one third of the T1DM group, respectively. On the other hand, as a ligand for TLR4, Fetuin-A has been proposed to promote adipogenesis by increasing triglyceride uptake into adipocytes, contributing to obesity, adipocyte dysfunction, and insulin resistance [2]. Excess fat in adipose tissue triggers fetuin-A expression by adipocytes. In fact, Fetuin-A mRNA expression has been detected only after prolonged adipocyte differentiation in obesity and has been suggested to exert local autocrine/paracrine or possibly even endocrine effects [20]. Since fetuin-A has been reported to mediate metabolic crosstalk between the fatty liver and the fatty pancreas by enhancing its secretion from pancreatic beta cells, these findings collectively suggest that, although the capacity of adipose tissue to produce fetuin-A remains uncertain [21], any contribution from this tissue may be regulated by circulating liver-derived fetuin-A. This underscores the role of this hepatokine in liver-adipose tissue cross-talk.

In our study, the use of FibroScan^®^, which provides a reliable estimate of the degree of steatosis and fibrosis [4], demonstrated the ability of fetuin-A to discriminate steatosis, but also showed its higher predictive ability for steatosis. However, no significant differences in fetuin-A levels were found between individuals with/without fibrosis, and no correlation between this hepatokine and liver fibrosis was observed. These results confirm and extend previous data suggesting fetuin-A as a potential early predictor of MASLD in individuals with centripetal obesity, although its relationship with fibrosis remains controversial [23]. Data have shown that its transcript expression is only barely detectable and remains unchanged during model aggravation, likely reflecting progressive accumulation in adipose tissue as liver disease progresses and suggesting no significant changes in fetuin-A levels with increasing disease severity [22, 24, 25]. Therefore, these findings further confirm fetuin-A as a potential circulating marker of liver steatosis, while its role as a prognostic factor needs to be explored in further studies [3].

The role of fetuin-A in T2DM remains unclear [11], with studies reporting both an increased risk of disease development [26–28] and no significant differences in established T2DM [21, 29]. The trend toward lower fetuin-A levels in the T2DM group than in those without T2DM and the negative association between T2DM and circulating fetuin-A found in our study, although not significant, confirm the need for further investigation. In particular, the clinical, anthropometric, and liver similarity between the T2DM/non-T2DM groups with obesity makes it difficult to distinguish the specific effects of obesity and T2DM.

A possible explanation may be a reduction in fetuin-A levels due to glucose- and lipid-lowering therapies (e.g., metformin, GLP-1 receptor agonists, statins) [11, 29–32]. In our cohort, these pharmacotherapies were significantly higher in the T2DM group than in the non-T2DM group with obesity, especially for metformin (Table S2). Furthermore, among all therapies, only the metformin-treated T2DM group had lower fetuin-A levels than the untreated group (Figure S3). Although not significant, these findings support the hypothesis that metformin treatment may attenuate the expected increase in fetuin-A [24, 33], as observed with high-dose metformin [24], common in T2DM.

Otherwise, the site of insulin resistance has been shown to influence fetuin-A levels [2], and increased levels of fetuin-A have been found in the adipose tissue of individuals with obesity and T2DM compared to those without T2DM [21]. The identification of T2DM as a cluster associated with the highest degree of liver and visceral fat [21], together with the consideration of adipose tissue as a “protective sponge”, may also explain why lower fetuin-A is observed in more advanced liver injury or in more severe liver disease [22, 34, 35]. In our study, the T2DM group had significantly higher levels of GGT and LSM and a higher percentage of LSM indicating advanced liver fibrosis (LSM ≥ 7.9 kPa). Therefore, it can be hypothesized that the coexistence of obesity and diabetes in the T2DM group may have triggered a compensatory mechanism that have contributed to lower fetuin-A levels. However, further studies with larger numbers of subjects are needed to confirm these findings.

Fetuin-A has been linked to liver insulin resistance in T1DM mouse model [6] and associated with disease duration, adiposity, poor glycemic control, and early complications, including early atherosclerosis and hepatosteatosis [7, 36]. Its elevated levels in patients with T1DM [7, 36], particularly those with MASLD [7] support its role as a potential biomarker in these patients. Emerging evidence suggests that increasing MASLD in T1DM reflects higher levels of overweight and obesity and MetS [37], defining a “double diabetes” phenotype [38]. We also recently found an independent association between poor glycemic control and obesity with steatosis, but not fibrosis, in T1DM patients with MASLD assessed by FibroScan^®^. These results highlight the potential role of both hyperglycemia and obesity in the pathogenesis of liver steatosis in these patients [37]. In our study, the T1DM group had the highest percentage of overweight, especially visceral adiposity, and poor glycemic control. FibroScan^®^ also detected liver steatosis in more than 1/3 of the patients despite normal liver enzymes. The predominant production by the liver and the possible limited contribution of adipose tissue, combined with the involvement of BMI, visceral adipose tissue and glucose levels in increasing fetuin-A levels [2] may explain the independent association found between fetuin-A and T1DM and the higher levels found in this group than in controls.

As the clinical value of non-invasive screening tools in T1DM remains under investigation [39], and that the diagnostic utility of serum liver enzyme levels is still limited [4], the global increase in these diseases and their complications highlights the urgent need for more accurate and standardized diagnostic strategies [40]. Recent evidence has further confirmed the limited performance of FIB-4 in patients with diabetes [4, 41]. In our study, we observed that even when age-specific cut-offs were applied, these thresholds tended to overestimate liver fibrosis, particularly in patients with T1DM, compared to FibroScan^®^ measurements, suggesting that caution is warranted with non-invasive liver assessment tools in this subgroup. Although clinical studies on this topic are still very limited, these results suggest fetuin-A as a reliable potential non-invasive for clinical assessment to evaluate liver health and metabolic dysfunction in these patients, pending the future establishment of reference values.

Finally, the positive correlation of fetuin-A with age is consistent with evidence recognizing age as a factor influencing its levels in liver steatosis [42], but with controversial results [34, 43, 44]. Although human studies have shown inconsistent results [45–47], the negative correlation of fetuin-A with male sex supports mouse models suggesting increased levels in females [48], probably influenced by sex hormones [49, 50]. Given that the women in our study are around postmenopausal status, it can be hypothesized that higher fetuin-A levels may be partly explained by the increased risk of MASLD and adiposity after estrogen loss protection during this period [51]. However, further studies are needed to explore these associations.

Study limitations include the small sample size and the use of TE rather than liver biopsy for the diagnosis of MASLD. Although the use of z-scores is not directly informative and does not correspond to specific biological or clinical cut-off values for biomarkers, it is consistent with previous studies that have recognized it as an effective novel approach for data harmonization [15, 16] overcoming the limitations of literature studies reporting inconsistent values that preclude direct comparison. Strengths include a well-described cohort in terms of clinical, anthropometric, biochemical and non-invasive liver assessment, including FibroScan^®^. In addition, no other studies have investigated fetuin-A in different metabolic disorders, including T1DM, compared to controls. These findings also support further studies to explore the role of fetuin-A in prognosis in the advanced liver disease cohort and in T2DM in treatment-naive patients.

## Conclusion

In conclusion, the correlation of fetuin-A with MetS features such as total and visceral anthropometric measurements, triglycerides and non-invasive liver assessment and its elevated levels in MASLD and metabolic disorders, including obesity, T2DM, T1DM suggests that fetuin-A mediates the cross-talk between liver and adipose tissue. Furthermore, routine measurement of this protein in serum supports its potential as a surrogate biomarker that could be included in multi-marker panels for early diagnosis and monitoring of hepatic steatosis and metabolic diseases. Further studies are needed to reach consensus on reference values and a true understanding of its biological significance, especially in T2DM, and its role as a prognostic factor, which is still limited.

## Supplementary Information

Below is the link to the electronic supplementary material.

Supplementary Material 1

## Data Availability

The data that support the findings of this study are available from the corresponding author upon reasonable request.
