# Supplementary material for: Fetuin-A: a potential molecular link between obesity, diabetes (type 2 and type 1) and metabolic steatotic liver disease (MASLD)
Source: J Endocrinol Invest. 2026 May 4;49(8):2033–43. doi: 10.1007/s40618-026-02880-w (PMC13486043; doi:10.1007/s40618-026-02880-w)
Supplement: Supplementary file 1 [file 40618_2026_2880_MOESM1_ESM.docx]

| **Characteristic** | **CTRL**  N = 13^1^ | **Obesity without DM**  N = 38^1^ | **T2DM**  N = 30^1^ | **T1DM**  N = 37^1^ | **p-value**^2^ |
| --- | --- | --- | --- | --- | --- |
| Sex (Male) | 3 (23.1%) | 8 (21%) | 9 (30%) | 16 (43%) | 0.12 |
| Age (Years) | 45 (34, 50) | 59 (55, 67) | 63 (59, 67) | 50 (40, 56) | **<0.001** |
| BMI (kg/m^2^) | 23 (21, 25) | 34 (32, 39) | 34 (31, 37) | 25 (23, 28) | **<0.001** |
| Obesity | 0 (0%) | 38 (100%) | 27 (90%) | 4 (11%) | **<0.001** |
| Wc (cm) | 74 (71, 79) | 112 (105, 121) | 116 (105, 119) | 87 (79, 96) | **<0.001** |
| Wc  (sex-specific cut-off) | 0 (0%) | 38 (100%) | 30 (100%) | 20 (54%) | **<0.001** |
| WHtR | 0.46 (0.44, 0.49) | 0.68 (0.65, 0.72) | 0.69 (0.65, 0.71) | 0.51 (0.48, 0.57) | **<0.001** |
| WHtR ≥ 0.5 | 0% | 38 (100%) | 30 (100%) | 20 (54%) | **<0.001** |
| AST (IU/L) | 18 (16, 20) | 21 (17, 24) | 21 (19, 27) | 21 (17, 27) | 0.2 |
| ALT (IU/L) | 14 (12, 19) | 21 (16, 25) | 22 (18, 29) | 19 (13, 24) | **0.009** |
| GGT (IU/L) | 15 (12, 18) | 24 (19, 28) | 32 (22, 46) | 18 (12, 27) | **<0.001** |
| Total cholesterol (mg/dL) | 180 (176, 187) | 197 (165, 220) | 166 (149, 192) | 157 (131, 176) | **<0.001** |
| HDL (mg/dL) | 71 (55, 80) | 53 (46, 61) | 50 (43, 56) | 60 (50, 72) | **0.001** |
| Triglycerides (mg/dL) | 60 (54, 74) | 128 (93, 162) | 127 (103, 152) | 63 (52, 80) | **<0.001** |
| LDL (mg/dL) | 103 (91, 122) | 110 (92, 143) | 90 (73, 121) | 78 (69, 93) | **<0.001** |
| Blood glucose (mg/dL) | 86 (80, 98) | 92 (85, 98) | 103 (92, 128) | 119 (97, 160) | **<0.001** |
| HbA1c (%) | 5.5 (5.3, 6.2) | 5.9 (5.5, 6.0) | 6.4 (5.9, 7.2) | 7.7 (6.8, 8.1) | **<0.001** |
| Insulin (µIU/mL) | 10.6 (10.6, 10.6) | 10.5 (8.0, 17.4) | 12.6 (8.5, 17.5) | NA (NA, NA) | 0.8 |
| Creatinine (mg/dL) | 0.83 (0.69, 0.93) | 0.82 (0.70, 0.93) | 0.83 (0.72, 0.96) | 0.79 (0.70, 0.95) | >0.9 |
| eGFR MDRD (mL/min) | 92 (66, 105) | 83 (69, 93) | 79 (72, 90) | 93 (87, 101) | 0.9 |
| eGFR CKD-EPI (mL/min) | 82 (71.7,92.6) | 86 (76.3,91.3) | 70 (62.9.79.0) | 74 (69.1,83.8) | 0.7 |
| Uric acid (mg/dL) | 5.90 (5.00, 6.80) | 5.35 (4.50, 6.50) | 5.75 (4.40, 6.90) | NA (NA, NA) | 0.8 |
| Albuminuria (mg/gr) | 9 (1, 17) | 6 (4, 22) | 8 (4, 14) | NA (NA, NA) | >0.9 |
| FLI | 5 (2, 11) | 89 (79, 95) | 89 (78, 96) | 13 (6, 46) | **<0.001** |
| FIB-4 | 1.08 (0.90, 1.22) | 1.39 (1.13, 1.61) | 1.42 (1.21, 1.67) | 1.29 (0.91, 1.53) | **0.004** |
| FLI ≥ 60 | 0% | 37 (100%) | 28 (100%) | 18 (56%) | **<0.001** |
| FIB-4 ≥ 1.3 (or ≥ 2.0 if age > 65 years) | 0% | 14 (36.8%) | 10 (33.3%) | 14 (37.8%) | **<0.001** |
| CRP (mg/dL) | 0.05 (0.03, 0.60) | 0.31 (0.21, 0.80) | 0.30 (0.14, 0.54) | 0.29 (0.12, 0.46) | 0.3 |
| Fetuin-A (z-scores) | -1.43 (-1.96, -1.07) | 0.71 (0.20, 1.21) | 0.13 (0.27, 0.74) | -0.18 (-0.95, 0.51) | **<0.001** |
| CAP (dB/m) | 176 (166, 194) | 293 (271, 318) | 284 (272, 303) | 213 (174, 248) | **<0.001** |
| CAP ≥ 248 dB/m | 0 (0%) | 34 (89.5%) | 28 (93.3%) | 13 (35.1%) | **<0.001** |
| LSM (kPa) | 3.90 (3.40, 4.20) | 5.55 (4.30, 6.40) | 6.55 (5.20, 7.40) | 4.70 (4.00, 5.90) | **<0.001** |
| LSM ≥ 7.9 kPa | 0 (0%) | 3 (8.6%) | 7 (23.3%) | 0 | **<0.001** |
|  |  |  |  |  |  |
| ^1^Median (Q1, Q3); n (%) |  |  |  |  |  |
| ^2^Kruskal-Wallis rank sum test; Pearson's Chi-squared test |  |  |  |  |  |

***Table S1*** *General characteristics of the whole population by group.*

*Continuous variables are reported as median (Q1, Q3) and compared using the Kruskal-Wallis test; categorical variables are reported as n (%) and compared using Pearson's χ² or Fisher's exact test, as appropriate. P values refer to global comparisons between groups. NA indicates data not available.*

*Abbreviations: CTRL, controls; T2DM, type 2 diabetes mellitus; T1DM:, type 1 diabetes mellitus; BMI, body mass index; WC, waist circumference; WHtR, waist-to-height ratio; AST, aspartate aminotransferase; ALT, alanine aminotransferase; GGT, γ-glutamyl transferase; HDL, high-density lipoprotein; LDL, low-density lipoprotein; HbA1c, glycated hemoglobin; eGFR, estimated glomerular filtration rate; MDRD, Modification of Diet in Renal Disease; CKD-EPI,* *Chronic Kidney Disease Epidemiology Collaboration; FLI, Fatty Liver Index; FIB-4, Fibrosis-4; CRP, C-reactive protein; CAP, controlled attenuation parameter; LSM, liver stiffness measurement.*
